# Efficacy and Safety of Ablative Fractional Laser-Assisted Delivery of Methotrexate in Adults with Localized Scleroderma: A Randomized and Controlled Clinical Trial

**DOI:** 10.3390/pharmaceutics14112261

**Published:** 2022-10-22

**Authors:** Qing Guo, Mingjie He, Junjie Cen, Danqi Huang, Shaoyun Hao, Zengqi Tang, Hui Xiong

**Affiliations:** 1 Department of Dermatology, Sun Yat-sen Memorial Hospital, Sun Yat-sen University, Guangzhou 510120, China; 2 Department of Dermatology, The Third Affiliated Hospital of Sun Yat-sen University, Guangzhou 510630, China; 3 Department of Chemical Pathology, The Chinese University of Hong Kong, Shatin New Territories, Hong Kong 999077, China; 4 Department of Ultrasonography, Sun Yat-sen Memorial Hospital, Sun Yat-sen University, Guangzhou 510120, China; 5 Guangdong Provincial Key Laboratory of Malignant Tumor Epigenetic and Gene Regulation, Sun Yat-sen Memorial Hospital, Sun Yat-sen University, Guangzhou 510120, China

**Keywords:** ablative fractional laser, methotrexate, laser-assisted drug delivery, localized scleroderma, morphea

## Abstract

Localized scleroderma (LS) is an autoimmune disease with sclerosis of the skin as the main manifestation. Currently, there is no specific treatment for LS. The effectiveness of ablative fractional laser (AFL) therapy for LS has been demonstrated in several studies. Combining ablative fractional Er:YAG laser therapy with topical methotrexate may yield therapeutic benefits for patients with LS. To compare the efficacy and safety of AFL-assisted delivery of methotrexate in adults with LS, we randomly divided patients into an AFL therapy group and an ablative fractional laser-assisted delivery of methotrexate (AFL+MTX) therapy group. Laser and assisted drug delivery treatment were given every four weeks for four months, and 22 patients completed the trial. Ultrasound measurements of dermal thickness and histological fibrosis degree and the Localized Scleroderma Cutaneous Assessment Tool (LoSCAT) score were used to assess therapeutic effects. Treatment results showed that both AFL and AFL-assisted methotrexate delivery were effective in treating LS, and the laser combined with methotrexate therapy was more effective in improving clinical appearance (*p* value = 0.042) and dermal thickness (*p* value = 0.016). No serious adverse reaction occurred in either group. In conclusion, AFL and assisted delivery of methotrexate are effective and safe treatments for LS.

## 1. Introduction

Localized scleroderma (LS), also known as morphea, is an autoimmune disease with sclerosis of the skin as the main manifestation, which can involve subcutaneous tissue, including the fat tissue, fascia, muscle and bone, but does not involve internal organs [1]. The incidence of LS is around 4 to 27 per 1 million people [2]. The proportion of men to women is reported at 1:2.6–6 [3,4]. LS is divided into five subtypes: plaque type, generalized type, line type, deep type and mixed type (a combination of two or more of these subtypes). Plaque and generalized type are much more prevalent in adults, and linear type is more common in children [5]. Although not life-threatening, LS has a considerable impact on the psychological state and appearance of patients. Therefore, the treatment of LS is necessary. Various methods have been tried to treat LS, including topically applied corticosteroids, calcineurin inhibitors and ultraviolet (UV) phototherapy. Systemic therapy of methotrexate (MTX) and/or corticosteroids are required during the acute phase of disease or when the subcutaneous tissues are involved [1,6]. High-dose MTX (doses ≥ 1 g/m^2^) is an antineoplastic drug used to treat patients with leukemia or lymphoma. Low-dose MTX (5–25 mg/week) is a commonly used disease-modifying antirheumatic drug in rheumatism. Multiple mechanisms of MTX potentially contribute to treating LS, including anti-inflammatory action, antifibrosis action [7] and regulating the expression of certain kinds of cytokines [8]. Some mechanisms may contribute to the anti-inflammatory actions of methotrexate, including adenosine release, uncoupling of nitric oxide synthases, increasing production of reactive oxygen species, activation of JUN N-terminal kinases (JNKs), increasing expression of long intergenic non-coding RNA p21 (lincRNA-p21), polyamine inhibition, direct or indirect inhibition of the Janus kinase-signal transducer, and activation of the transcription (JAK–STAT) signaling pathway and the nuclear factor-κB (NF-κB) signaling pathway [9,10,11]. A study showed that the anti-inflammatory and immunosuppressive actions of low-dose MTX are mainly achieved by inhibiting the JAK-STAT signaling pathway [12]. The anti-fibrogenic effect of methotrexate may be attributed to its anti-inflammatory effect or its direct action on fibroblasts in the dermis. Two studies on adult LS and adult systemic sclerosis support the antifibrotic effect of MTX [13,14]. MTX is a strong immunosuppressant that can inhibit both lymphocyte-mediated and antibody-mediated immune responses by reducing lymphocyte migration in the skin and modulating intracellular and extracellular signaling [15]. Moreover, MTX suppresses the secretion of various cytokines, including TNF-a, IL-6 and IL-8 [16]. Unfortunately, topical treatments are ineffective and systemic treatments have a variety of adverse effects for some patients. Recently, with the development of ablative fractional laser (AFL), some researchers have tried using AFL to treat LS. Shalaby et al. conducted a controlled trial of 17 LS patients using UVA-1 phototherapy (30 J/cm^2^) and CO_2_ fractional laser therapy (wavelength of 10,600 nm, power of 25 W) and demonstrated that compared with low-dose UVA-1 phototherapy, patients treated with CO_2_ fractional laser showed obvious improvements in clinical, histopathological and ultrasonographic examinations [17].Up to now,, AFL has assisted the delivery of more than 70 drugs through the skin [18]. The advantages of laser-assisted drug delivery (LADD) include high local drug concentration, low drug concentration in the blood and no drugs passing through the gastrointestinal tract. Therefore, compared with systemic drug delivery, LADD is safe without liver and kidney damage. It has been found that AFL-assisted drug delivery is superior to other physical methods such as curettage, microdermabrasion, microneedles and non-ablative fractional laser [19]. Methotrexate is a first-line treatment option for LS with alopecia, nausea, headache, fatigue and hepatotoxicity when used systemically. We believe that AFL-assisted delivery of methotrexate is effective in improving LS while avoiding adverse effects. To our knowledge, there are no studies thus far on AFL-assisted delivery of methotrexate for the treatment of LS. We are hopeful that AFL-assisted delivery of methotrexate will be a new way of treating LS.

## 2. Materials and Methods

The study was designed as a two-armed, randomized, double-blind, comparative trial. The study was approved by the Institutional Review Board of Sun Yat-sen Memorial Hospital of Sun Yat-sen University (2019-KY-089) and was conducted in accordance with the 1975 Declaration of Helsinki. The trial was registered in the Chinese Clinical Trial Register (registration number: ChiCTR2000031572).

### 2.1. Patients

**Figure 1:**
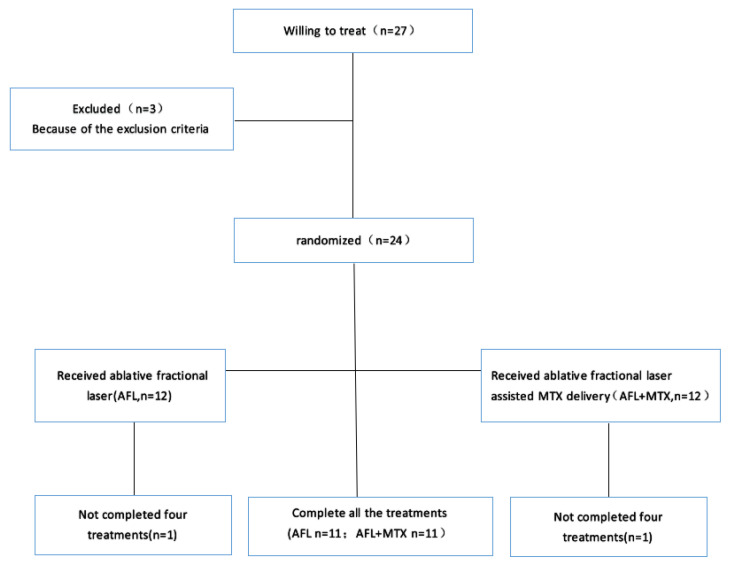
Flow diagram of the clinical trial. AFL group was treated by ablative fractional laser; AFL+MTX group was treated by ablative fractional laser-assisted delivery of methotrexate.

Between January 2019 and June 2022, patients aged ≥18 years who had biopsy-confirmed LS were enrolled in this study. Inclusion criteria were as follows: (1) patients met LS diagnostic criteria [6]; (2) patients were newly diagnosed or without any LS treatment in the past 4 weeks. Exclusion criteria were as follows: (1) lactating or pregnant women; (2) patients were allergic to the MTX solution or lidocaine cream; (3) patients had a tendency of keloid formation; (4) patients had other serious diseases such as liver, kidney or heart damage; (5) patients were unable to complete four treatments for any reason. A total of 27 patients of LS were willing to be treated, 3 patients were excluded and 2 patients dropped out (Figure 1). In the end, 22 patients completed the clinical trial. All the patients gave informed consent, and general information was recorded before treatment (Table 1).

### 2.2. Treatment

Patients were randomly divided into two groups. The AFL group was treated by ablative fractional laser and the AFL+MTX group was treated by ablative fractional laser-assisted delivery of methotrexate. Ablative fractional laser was performed using a 2940 nm Er:YAG fractional laser (Sciton, Palo Alto, CA, USA) at 600 μm ablation depth, level 1 coagulation, 11% treatment density and a single pulse. For the AFL+MTX group, immediately after AFL, 1 ml of 20 mg/mL MTX solution (Pfizer Inc., New York, NY, USA) was applied to the treatment region (1% body surface area). These lesions were covered with an occlusive dressing for 5 h. For the AFL group, the lesions were covered with 1 mL of 0.9% NaCl solution and an occlusive dressing for the same amount of time. The treatment was every four weeks, amounting to 4 times in total.

### 2.3. Clinical Evaluation

The Localized Scleroderma Cutaneous Assessment Tool (LoSCAT) score was evaluated by two dermatologists 4 weeks before and after treatment. LoSCAT is an instrument using simple clinical examination to quantify disease damage and activity. The total score of LoSCAT contains modified Localized Scleroderma Skin Activity Index (mLoSSI) and Localized Scleroderma Skin Damage Index (LoSDI) scores. The mLoSSI score is used to describe new or enlarged LS lesions, erythema and skin thickness. No new or enlarged LS lesion indicates grade 0, and a new or enlarged LS lesion indicates grade 3. Erythema and skin thickness are graded from 0 to 3 and then summed to obtain the mLoSSI score. The LoSDI score is used to describe the presence and extent of skin damage, dermal atrophy (DAT), subcutaneous atrophy (SAT) and dyspigmentation (DP) in LS lesions at 18 cutaneous anatomic sites. DAT, SAT and DP are graded from 0 to 3 and then summed to obtain the LoSDI score [20].

### 2.4. Ultrasound and Histopathological Evaluation

A 5–18 MHz ultrasound (Cannon, Tokyo, Japan) was used to measure the dermal thickness of the treatment area, and a pathological biopsy was performed 4 weeks before and after the treatment. The biopsy samples were fixed, sectioned into 5 μm thick slices and stained with hematoxylin and eosin (H&E). Two pathologists used standard light microscopy to evaluate the fibrosis extent of H&E-stained sections. According to the extent of fibrosis, the degree of histological fibrosis has three increasing grades (grade 1 to 3). Compared to normal controls, the three increasing grades of fibrosis were defined as follows: (i) grade 1 = weak fibrosis, defined as no fibrosis in the papillary dermis and light fibrosis in the superficial reticular dermis or in the median reticular dermis or in the deep reticular dermis; (ii) grade 2 = moderate fibrosis, defined as not belonging to grade 1 or 3; and (iii) grade 3 = severe fibrosis, defined as severe fibrosis in the deep reticular dermis and in the median reticular dermis no matter the extent of fibrosis in the superficial reticular dermis and in the papillary dermis, or severe fibrosis in the deep reticular dermis or moderate fibrosis in the median reticular dermis, superficial reticular dermis and the papillary dermis [21].

### 2.5. Safety Assessment

Patients’ blood was drawn to examine routine blood cell analysis, liver and kidney functions, and circulating autoantibodies four weeks before and after treatment. Patients completed the Dermatology Quality of Life Index (DLQI) questionnaire and gave satisfaction scores at the end of the trial. The scores of patient satisfaction were documented as very satisfied (3), satisfied (2), slightly satisfied (1) or dissatisfied (0).

### 2.6. Statistical Analysis

All statistical analyses were conducted with SPSS version 25.0 (SPSS Inc., Chicago, IL, USA). Data following a normal distribution are presented as mean ± standard deviation or otherwise expressed as median and inter-quartile range. For comparison between paired parameters (before and after treatment), the choice of paired-sample *t*-test or Wilcoxon matched-pairs signed-rank test depends on the normality of the data. For comparison between independent groups, the choice of independent samples *t*-test or rank sum test depends on the normality of the data. Values of *p* < 0.05 were considered statistically significant.

## 3. Results

### 3.1. Clinical Improvement

**Figure 2:**
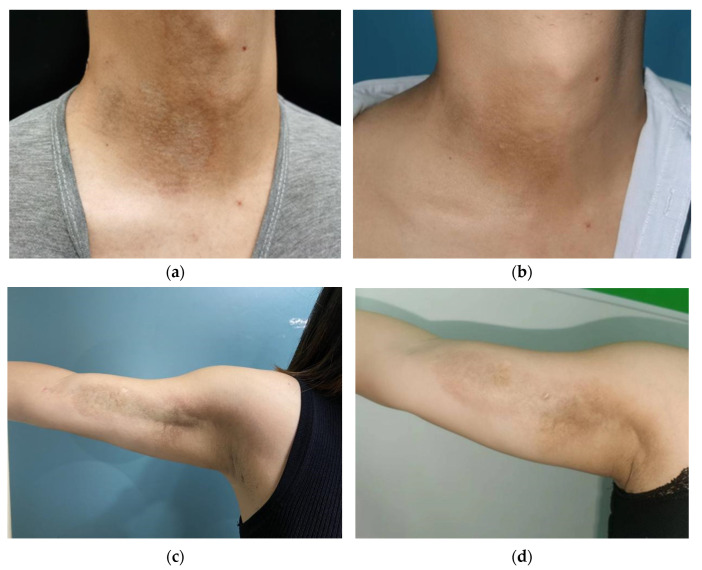
Clinical photography of patients. (**a**) Patient 4 weeks before treatment with ablative fractional laser-assisted delivery of methotrexate. (**b**) Patient 4 weeks after treatment with ablative fractional laser-assisted delivery of methotrexate. (**c**) Patient 4 weeks before treatment with ablative fractional laser. (**d**) Patient 4 weeks after treatment with ablative fractional laser.

Before treatment, the lesions were ivory-white or yellow-white sclerotic plaques with a rough, dry or smooth, waxy luster surface and leathery hardness when touched, mostly accompanied by capillary dilation and surrounding pigmentation. After treatment, the lesion area was reduced, and the border with normal skin was not clear. The color of the lesion was light red or light white. The hardness of lesions was softer than before when touched, and the degrees of pigmentation and capillary dilation were less than before (Figure 2).

Based on the scores adopted and modified from LoSCAT, compared to before treatment, the two treatment groups had significant improvements in the clinical scores. The median LoSCAT score was 9 before treatment (inter-quartile range: 8–14) and 8 after treatment (inter-quartile range: 6–14) in the AFL group, indicating that Er:YAG fractional laser treatment of LS can reduce disease activity and improve disease severity (*p* value < 0.05). The median LoSCAT score was 11 before treatment (inter-quartile range: 9–15) and 4 after treatment (inter-quartile range: 4–11) in the AFL+MTX group, indicating that Er:YAG fractional laser-assisted delivery of methotrexate treatment of LS helped to reduce disease activity and improve disease severity (*p* value < 0.01). The AFL+MTX group was superior to the AFL group in clinical improvement (*p* value < 0.05) (Table 2).

### 3.2. Ultrasound Assessment of Dermal Thickness

**Figure 3:**
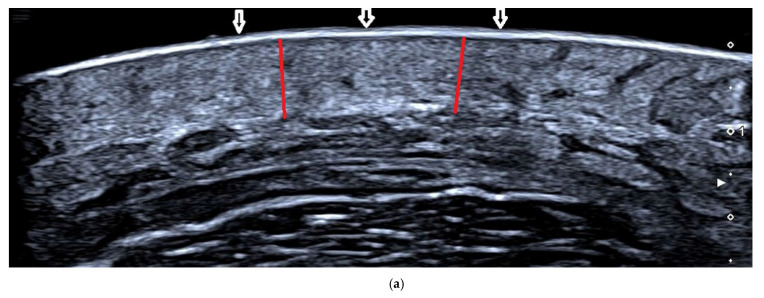

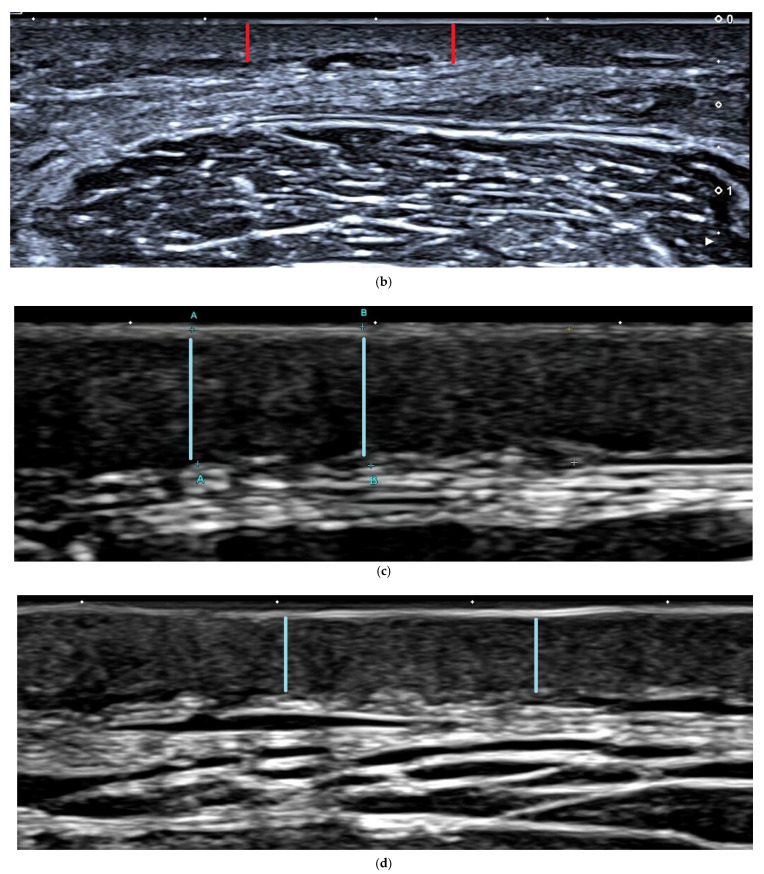
Ultrasonic images of patients four weeks before and after treatment. (**a**) AFL+MTX group patient 4 weeks before treatment. The uppermost layer, the epidermis layer, is hyperechoic (shown with white arrows), and the underlying dermis is hypoechoic (shown with red line) and indistinguishable from the fat layer. (**b**) AFL+MTX group patient 4 weeks after treatment. The dermis is thinner than before (shown with red line) and can be easily distinguished from the fat layer. (**c**) AFL group patient 4 weeks before treatment. The dermis (shown with blue line) is indistinguishable from the fat layer. (**d**) AFL group patient 4 weeks after treatment. The dermis is thinner (shown with blue line) than before and clearly identified with the fat layer.

A statistically significant decrease in dermal thickness after treatment was noted compared to before treatment in both groups (both *p* value = 0.001). Comparing the two groups, the AFL+MTX group had superior decreased dermal thickness than the AFL group (*p* value = 0.016) (Table 3) (Figure 3).

### 3.3. Histopathological Results

**Figure 4:**
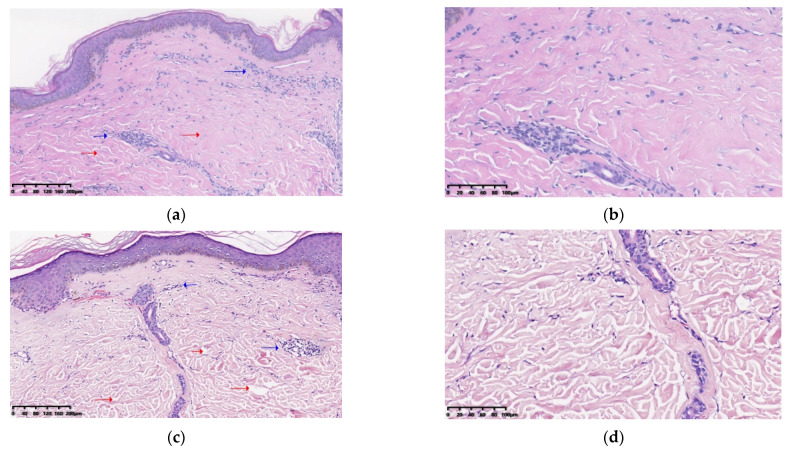

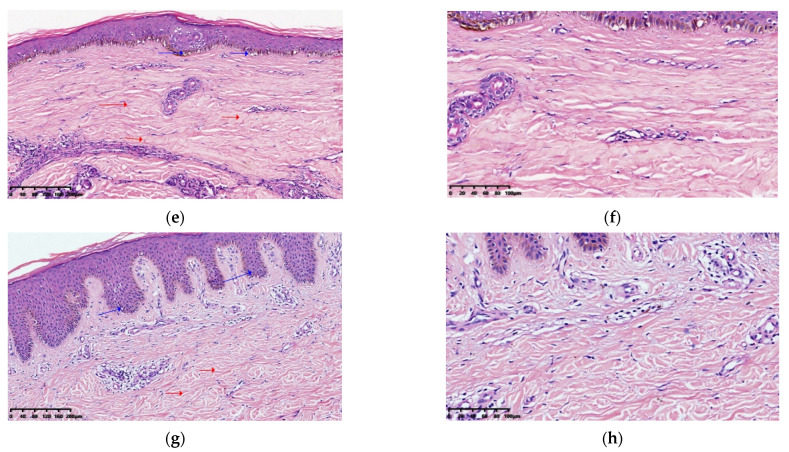
The microscopic appearance of a patient’s H&E-stained sections in the treatment region. (**a**) (×100) and (**b**) (×200) are the H&E-stained sections of a patient in the AFL+MTX group before treatment. Dense collagen deposition is shown with red arrows. Perivascular and periappendageal moderate admixed inflammatory cell infiltration is shown with blue arrows. (**c**) (×100) and (**d**) (×200) are the H&E-stained sections of the patient one month after four treatments. Decreased collagen deposition and a widened collagen gap are shown with red arrows. Mild admixed inflammatory cell infiltration is shown with blue arrows. (**e**) (×100) and (**f**) (×200) are the H&E-stained sections of a patient in the AFL group before treatment. Epidermal atrophy and vacuolar degeneration of the basal cell layer are shown with blue arrows. Thickish collagen fibers and a narrow collagen gap are shown with red arrows. (**g**) (×100) and (**h**) (×200) are the H&E-stained sections of the patient one month after four treatments. Thicker epidermis and marked elongation rete ridges are shown with blue arrows. Thinner collagen fibers and a wider collagen gap are shown with red arrows.

Before treatment, dense collagen is deposited in the dermis, and perivascular and periappendageal moderate admixed inflammatory cells infiltrate the dermis (shown in Figure 4a,b,e,f). After treatment, there is decreased collagen deposition, a widened collagen gap and thinned dermis compared to before treatment (shown in Figure 4c,d,g,h). In addition, epidermal atrophy and vacuolar degeneration of the basal cell layer can be observed before treatment (shown in Figure 4e,f). After treatment, the epidermis is thicker and rete ridges are more elongated than before (shown in Figure 4g,h). For the AFL group, five patients were classified in the grade 2 subgroup with moderate fibrosis and six patients in the grade 3 subgroup with severe fibrosis before treatment. For the AFL+MTX group, the grade of the histological fibrosis degree was the same as the AFL group before treatment. After treatment, the grade 1 subgroup with mild fibrosis had two patients in the AFL group and four patients in the AFL+MTX group. The grade 2 subgroup with a moderate degree of fibrosis had eight patients in the AFL group and seven patients in the AFL+MTX group. The grade 3 subgroup with severe fibrosis had one patient in the AFL group and none in the AFL+MTX group. Statistical differences were found before and after treatment in AFL and AFL+MTX groups (both *p* value < 0.05) (Table 4, Figure 4).

### 3.4. Safety Analysis

Adverse reactions after treatment mainly included temporary pain, redness, swelling, crusting and hyperpigmentation. The pain, redness and swelling usually lasted 1 to 4 days. The crusts fell off within 5 to 10 days. Hyperpigmentation gradually faded within 3 months. No serious adverse event, including infection or scarring, occurred in either group during treatment. For the AFL group, the average DLQI score is 3.91 ± 5.15 and the mean satisfaction score is 1.73 ± 1.19. For the AFL+MTX group, the average DLQI score is 6.36 ± 5.46 and the mean satisfaction score is 2.18 ± 0.87. There is no significant difference between the two treatment groups (*p* value > 0.05) (Table 5). There are no obviously abnormal results in blood cell analysis and liver and kidney function before and after treatment in both treatment groups. For circulating autoantibodies in 22 patients, 13 patients (59.1%) were ANA-positive. Two of the thirteen patients were positive for anti-SSA antibodies, one was anti-SSB antibody-positive, two were anti-ribosomal antibody-positive and one was anti-centromere B antibody-positive.

## 4. Discussion

The results of our trial show that both the erbium fractional laser and laser-assisted methotrexate delivery were effective in treating LS, and the laser combined with methotrexate therapy was more effective in improving clinical appearance (*p* value = 0.042) and dermal thickness (*p* value = 0.016). It is well known that the basic principle of AFL is photothermal action, in which multiple uniform laser channels are created instantaneously. The laser channels are called microthermal treatment zones (MTZs). AFL treats LS by promoting the degradation of abnormal collagen and inducing normal collagen synthesis. Its effectiveness has been demonstrated in several studies [17,22,23]. During the AFL-induced injury response, the MTZ area is immediately filled with many micro epidermal necrotic debris (MEND) and tissue exudate, and surrounded by a coagulated area of thermal injury. Heat shock proteins (HSPs), matrix metalloproteinases (MMPs), growth factors and other mediators are involved in injury repair. On the third day of laser treatment, there are increased HSPs; first is an increase in HSP72, stimulating the activation of epidermal stem cells and dermal cells, promoting rapid damage repair and collagen remodeling. HSP72 expression gradually decreases after 14 days. Four to seven days after laser treatment, HSP47 expression is upregulated and sustained for three months, resulting in increased accumulation of procollagen and collagen I and III. In addition, TGF-β1 gradually increases on day 3 of laser treatment and decreases over the next 30 days, promoting fibroblast proliferation and subsequent collagen deposition in the early stage [24,25]. MMPs such as 1, 3, 9 and 13 promote collagen degradation. MMP1 and MMP3 gradually increase over 3 days of laser treatment, peak at 7 days and decrease in 2 weeks. The levels of MMP 9 and MMP13 remain elevated for a longer period of time to promote residual collagen degradation [26]. MEND exfoliation is entirely complete within a month, and collagen III is replaced by collagen I. After approximately 3 months, MTZ is replaced by fresh collagen tissue. Similar to our findings, other studies found a reduction in dermal thickness of LS lesions in the early days after fractional laser treatment [17,27]. In addition to the direct therapeutic effect of the laser on the lesions, the MTZs increased the local concentration of methotrexate on the lesions. Due to the hydrophilic nature of methotrexate, it is difficult to penetrate the skin barrier. Chemical enhancement strategies and physical techniques such as iontophoresis, microneedles, diode laser and ablative fractional Er:YAG laser are used to enhance the local concentration of methotrexate [28,29,30]. Methotrexate has a mechanism of action similar to folic acid, inhibiting dihydrofolate reductase to prevent the reduction of dihydrofolate to active tetrahydrofolate. It inhibits the proliferation of cells by inhibiting DNA and RNA synthesis [13]. Although the mechanism of action of MTX in LS has not been fully elucidated, numerous studies have demonstrated that the efficiency of systemic use of MTX for clinical improvement of LS is more than 50%, both in pediatric and adult patients [31]. Methotrexate plays an important role as an anti-inflammatory drug in rheumatic diseases by inhibiting IL-1 and IL-6 [32]. Decreases in circulating IL-6 and soluble IL-2 have been reported with MTX [33,34]. Increased serum levels of IL2, IL-4 and IL-6 in patients with LS have been confirmed. The decrease in these cytokines is associated with an improvement in skin sclerosis [35]. We did not measure the concentration of methotrexate in the skin lesions after drug delivery. However, based on the superior trial results in the AFL+MTX group compared to the AFL group, we believe that the Er fractional laser-assisted MTX delivery increased the concentration of MTX on the lesions and that the topical high concentration of MTX worked together with the Er fractional laser on the LS. Two hours after AFL-assisted delivery of methotrexate therapy, we determined the content of methotrexate in some patients’ plasma, and it was too low to be detected. Therefore, in our trial, there were no common adverse effects of systemic methotrexate delivery, such as gastrointestinal discomfort, hepatic impairment, bone marrow suppression, etc. In our study, more than half of the patients showed temporary hyperpigmentation and gradually recovered to normal within three months. Although we chose the Er:YAG fractional laser instead of the CO_2_ fractional laser to reduce the occurrence of hyperpigmentation, the CO_2_ fractional laser is more likely to cause adverse reactions such as scarring and hyperpigmentation than the Er:YAG fractional laser. Therefore, we should be more careful in using the ablative fractional laser to treat LS. For laser-assisted drug delivery, it does not matter which laser is chosen, as we can set parameters such as energy and depth to make the Er:YAG fractional laser and the CO_2_ fractional laser produce the same size channel [18,36].

The shortcoming of the trial is that we had few patients because of the low morbidity of LS, which may influence the statistical results. Because of the limited number of studies on laser-assisted drug delivery and the different laser devices, the treatment parameters of fractional lasers are mainly based on the experience of the therapists. We conducted pre-experiments with the laser parameters, drug doses and drug concentrations in model mice before the trial. The results of the pre-experiments (shown in Supplementary Table S1) are consistent with the final experimental results. However, for laser-assisted methotrexate delivery to treat LS, the ideal laser parameters, drug dosage, drug concentration and frequency of treatment still require further study.

## 5. Conclusions

We systematically and comprehensively evaluated the therapeutic effects of AFL and assisted delivery of methotrexate for the treatment of LS in terms of clinical improvement, ultrasounds and histological results. AFL and assisted delivery of methotrexate are effective and safe for the treatment for LS.

## Figures and Tables

**Table 1:** Characteristics of included patients. The age and the duration of disease are presented as mean ± standard deviation, and the lesion areas of patients are expressed as median and inter-quartile range. AFL group was treated by ablative fractional laser and AFL+MTX group was treated by ablative fractional laser-assisted delivery of methotrexate.

Characteristics	AFL	AFL+MTX	*p* Value
Sex (*n*)	11	11	1.000
Male (*n*, %)	2 (18.2)	2 (18.2)	
Female (*n*, %)	9 (81.8)	9 (81.8)	
Age (mean ± SD), y	34.82 ± 13.31	34.09 ± 9.75	0.885
Disease type (*n*, %)			1.000
plaque type	7 (63.6)	6 (54.5)	
generalized type	2 (18.2)	2 (18.2)	
line type	2 (18.2)	3 (27.3)	
Disease stage (*n*, %)			0.586
edema stage	0 (0.0)	1 (9.1)	
sclerotic stage	8 (72.7)	9 (81.8)	
atrophic stage	3 (27.3)	1 (9.1)	
Total lesions (*n*)	2 (1–3)	2 (1–4)	0.906
Lesion area (cm^2^)	36.00 (18.00–64.00)	64.00 (35.00–154.00)	0.156
Duration (mean ± SD), y	12.40 ± 9.52	6.29 ± 3.94	0.070

**Table 2:** The LoSCAT scores four weeks before and after treatment. Data are expressed as median and inter-quartile range. Values of *p* < 0.05 are considered statistically significant. AFL group was treated by ablative fractional laser and AFL+MTX group was treated by ablative fractional laser-assisted delivery of methotrexate.

	AFL	AFL+MTX	*p* Value
Before	9 (8–14)	11 (9–15)	0.296
After	8 (6–14)	4 (4–11)	0.042
*p* value	0.041	0.005	/

**Table 3:** The dermal thickness four weeks before and after treatment. Data are expressed as median and inter-quartile range. Δd is the difference value of the dermal thickness four weeks before and after treatment. Data are expressed as mean ± standard deviation. AFL group was treated by ablative fractional laser and AFL+MTX group was treated by ablative fractional laser-assisted delivery of methotrexate.

Dermal Thickness (mm)	AFL	AFL+MTX	*p* Value
Before	1.723 (1.352–2.382)	2.084 (1.653–2.383)	/
After	1.589 (1.206–2.296)	1.800 (1.393–2.154)	/
Δd	0.138 ± 0.085	0.323 ± 0.205	0.016
*p* value	0.001	0.001	/

**Table 4:** The histological grade of fibrosis four weeks before and after treatment. AFL group was treated by ablative fractional laser and AFL+MTX group was treated by ablative fractional laser-assisted delivery of methotrexate.

Group	Treatment	*n*	Grade 1	Grade 2	Grade 3	*p* Value
AFL	Before	11	0	5	6	0.035
	After	11	2	8	1	
AFL+MTX	Before	11	0	5	6	0.004
	After	11	4	7	0	

**Table 5:** Adverse events, DLQI and satisfaction scores of the two treatment groups. AFL group was treated by ablative fractional laser and AFL+MTX group was treated by ablative fractional laser-assisted delivery of methotrexate.

		AFL (*n* = 11)	AFL+MTX (*n* = 11)	*p* Value
Pain	Number of patients	11	11	
	Duration (day)	1.64 ± 0.81	1.82 ± 0.75	0.618
Redness	Number of patients	11	11	
	Duration (day)	2.00 ± 0.632	2.09 ± 0.632	0.465
Crust	Number of patients	11	11	
	Decrustation (day)	7.82 ± 1.25	6.91 ± 1.30	0.110
Pigmentation	Number of patients	6	6	
	Duration (day)	34.00 ± 15.52	39.67 ± 20.63	0.603
Infection	Number of patients	0	0	
Scarring	Number of patients	0	0	
DLQI score		3.91 ± 5.15	6.36 ± 5.46	0.291
Satisfaction score		1.73 ± 1.19	2.18 ± 0.87	0.401

## Data Availability

Data supporting the reported results can be found in REDCap (https://www.redcap.gzsys.org.cn:8082, accessed on 9 August 2022.).

## Supplementary Materials

The following supporting information can be downloaded at: https://www.mdpi.com/article/10.3390/pharmaceutics14112261/s1. Table S1: The dermal thickness on left and right back of skin lesions of mice at 2 weeks after 4 treatments. The untreated left back skin lesions are used as a self-blank control (CT). The skin lesions treated with Er:YAG fractional laser-assisted delivery of drugs on the right back are the laser group (LS). Δd is the difference value of the dermal thickness of skin lesions between left and right back. Five mice in the blank control group (Group 1) were injected with 100 μL PBS solution on both sides of the back daily for three weeks. After that, Group 1 was treated with AFL-assisted delivery of MTX (dose of 1 mL, concentration of 20 mg/mL) and encapsulated for 5 h weekly for four weeks. Model groups of mice (Groups 2 and 3, five mice each) were injected subcutaneously with 100 μL of bleomycin solution (dissolved in PBS solution, 0.2 mg/mL) on both sides of the back daily for 3 weeks. After laser treatment, Group 2 was treated with 1 mL of 0.9% NaCl solution and Group 3 was treated with 1 ml of methotrexate solution (20 mg/mL) weekly for four weeks. Data are expressed as mean ± standard deviation. Values of *p* < 0.05 are considered statistically significant.
