# Supplementary material for: Efficacy and Safety of Ablative Fractional Laser-Assisted Delivery of Methotrexate in Adults with Localized Scleroderma: A Randomized and Controlled Clinical Trial
Source: Pharmaceutics. 2022 Oct 22;14(11):2261. doi: 10.3390/pharmaceutics14112261 (PMC9696356; doi:10.3390/pharmaceutics14112261)
Supplement: Supplementary file 1 [file pharmaceutics-14-02261-s001.zip › pharmaceutics-1936349-supplementary.pdf]

Supplemental data:

Supplemental Table S1. The dermal thickness on left and right back of skin lesions of mice at 2 weeks after 4 times treatments. The untreated left back skin lesions are used as self-blank control(CT). And the skin lesions treated with Er:YAG fractional laser assisted delivery of drug on the right back are laser group(LS). Δd is the difference value of the dermal thickness of skin lesions between left and right back. Five mice in Blank control group(group 1) were injected by the 100μl PBS solution on both sides of the back daily until three weeks. After that, Group 1 was treated with AFL assisted delivery of MTX(dose of 1ml, concentration of 20mg/ml) and encapsulated for 5 hours weekly until four weeks. Model groups of mice(group 2 and 3 , five mice, respectively) were injected subcutaneously by 100μl of bleomycin solution(dissolved in PBS solution, 0.2mg/ml)on both sides of the back daily until 3 weeks. After laser treatment, Group 2 was treated with 1ml of 0.9%NaCl solution and Group 3 was treated with 1ml of methotrexate solution(20mg/ml) weekly until four weeks. Data was expressed as average±standard deviation. Values of $p < 0.05$ were considered statistically significant.

**Dermal thicness(mm)**	**CT**	**LS**	**Δd**	**P value**
Group 1	0.532±0.031	0.527±0.015	0.031±0.018	0.801
Group 2	0.727±0.066	0.621±0.032	0.106±0.039	0.004
Group 3	0.720±0.033	0.618±0.091	0.102±0.078	0.043
